# Canine Visceral Leishmaniasis, United States and Canada, 2000–2003

**DOI:** 10.3201/eid1203.050811

**Published:** 2006-03

**Authors:** Zandra H. Duprey, Francis J. Steurer, Jane A. Rooney, Louis V. Kirchhoff, Joan E. Jackson, Edgar D. Rowton, Peter M. Schantz

**Affiliations:** * Centers for Disease Control and Prevention, Atlanta, Georgia, USA;; † University of Iowa, Iowa City, Iowa, USA;; ‡ Walter Reed Army Institute of Research, Washington, DC, USA

**Keywords:** Canine leishmaniasis, Foxhounds, Leishmania spp

## Abstract

Visceral leishmaniasis, caused by protozoa of the genus *Leishmania donovani* complex, is a vectorborne zoonotic infection that infects humans, dogs, and other mammals. In 2000, this infection was implicated as causing high rates of illness and death among foxhounds in a kennel in New York. A serosurvey of >12,000 foxhounds and other canids and 185 persons in 35 states and 4 Canadian provinces was performed to determine geographic extent, prevalence, host range, and modes of transmission within foxhounds, other dogs, and wild canids and to assess possible infections in humans. Foxhounds infected with *Leishmania* spp. were found in 18 states and 2 Canadian provinces. No evidence of infection was found in humans. The infection in North America appears to be widespread in foxhounds and limited to dog-to-dog mechanisms of transmission; however, if the organism becomes adapted for vector transmission by indigenous phlebotomines, the probability of human exposure will be greatly increased.

## Abstract 2

Foxhounds infected with *Leishmania* spp*.* were found in 18 states and 2 Canadian provinces.

Visceral leishmaniasis, caused by geographic variants of the *Leishmania donovani* complex (*L. donovani*, *L. infantum*, *L. chagasi*), is a progressive wasting disease of dogs and humans that is often fatal if untreated (*1*). Agents of the *L. donovani* complex occur in parts of Mediterranean Europe, the Middle East, Asia, Africa, and Central and South America (*1**–**3*). In infections involving the *L. donovani* complex in the Mediterranean region (*L. infantum*) and in South America (*L. chagasi*), dogs are reservoirs for human infection (*1**,**2*). Parasites are usually transmitted between hosts by phlebotomine sandflies (*Lutzomyia* or *Phlebotomus* spp.) (*2**,**3*).

Direct quantitative relationships between prevalence of leishmaniasis in local dog populations and incidence of human disease have been reported (*4*). Infection in dogs may indicate human risk for leishmaniasis, especially in HIV-positive persons, in many areas (*5*); infected but asymptomatic dogs can infect sandflies that feed on them, posing a risk to uninfected dogs and humans (*6*).

Until recently, visceral leishmaniasis was thought to be primarily an imported disease in North America; infected dogs had usually been imported from regions in southern Europe or South America where *L. infantum* and *L. chagasi* were enzootic (*2**,**3*). However, sporadic cases of leishmaniasis have been reported in foxhounds and dogs of other breeds with no history of travel to areas where leishmaniasis was enzootic, and the origin of these infections remains unknown (*7**,**8*).

In the late summer of 1999, foxhounds at a New York foxhunting club began showing signs of epistaxis, weight loss, muscle atrophy, seizures, alopecia, dermal lesions, swollen limbs and joints, and renal failure (*10*). Of the 250 dogs in the kennel, 112 (44.8%) were sick and 29 (11.6%) had died at the time of the investigation. Cytopathologic examination of joint fluid of 1 hound showed amastigote forms of *Leishmania* spp. These parasites were found at necropsy of several dogs by using indirect immunofluorescent assay (IIF), polymerase chain reaction, culture, and cytologic and histopathologic studies (*9*). At that time, autochthonous leishmaniasis had not been reported in dogs, other animals, or humans in New York.

Diagnostic surveys were initiated to measure the prevalence of *Leishmania* infection at the index hunt club and to determine how infection was introduced into and transmitted among these dogs; the investigation was extended to foxhounds, other breeds of dogs, and wild canids in other states. We describe the results of the 3-year investigation of canine visceral leishmaniasis in the United States and Canada through February 2003.

## Materials and Methods

The Masters of Foxhounds Association of America (MFHA) represents >200 foxhound kennels and hunt clubs that house >12,000 foxhounds in 35 US states and 4 Canadian provinces. In conjunction with MFHA and numerous state health departments, the Centers for Disease Control and Prevention (CDC) invited all MFHA-registered foxhound owners to participate in this investigation. Owners of nonregistered foxhound hunt clubs that were in close proximity to MFHA-registered hunt clubs were also invited to participate. Dog owners were asked to submit samples in 3- to 4-month intervals. From hounds identified as seropositive for *Leishmania* spp., bone marrow and other specimens were requested for parasitologic diagnosis. Serum samples from dogs of breeds other than foxhounds and from wild canids (e.g., foxes and coyotes) were also obtained and tested for antibodies to *Leishmania* spp. and *Trypanosoma cruzi*. Samples from other dog breeds were obtained from kenneled "pound dogs" in Virginia and New York, where infection in foxhounds had been identified, and from pet dogs whose sera were tested at CDC for *Leishmania* antibodies before the pets traveled to countries that require such testing. Fox and coyote samples were provided from animals trapped in various locations of the southeastern United States.

IIF assays for antibodies to *Leishmania* spp. were performed on human and canine serum samples submitted to CDC (*10*). IIF was considered positive when fluorescence was observed around the organisms on the slide. Fourfold dilutions were used to reach the final endpoint titer. The CDC standard IIF diagnostic cutoff titer for infection by *Leishmania* spp. belonging to the donovani complex in dogs is >128 (*10*).

To assess possible infection in humans, persons associated with dogs in the study were invited to submit serum samples for testing. After explaining the purpose of the study and obtaining informed consent, participants were asked about their contact with foxhounds and their personal health status. Serum samples were tested for *Leishmania* antibodies by using the same technique, with the same titer value for determining a positive reaction (*3**,**10*).

Other antemortem samples submitted from dogs included aspirates of spleen, liver, or lymph nodes and excisional lymph node biopsy specimens. Postmortem specimens submitted included blood, bone marrow, lymph nodes, kidney, spleen, liver, brain, testes, epididymis, ovaries, and neoplasms. Two media were used to culture *Leishmania* spp: Novy-MacNeal-Nicolle (NNN) medium with Offutt modification and modified NNN medium with Roswell Park Memorial Institute medium overlay (*11*).

*T. cruzi* antibodies cross-react and give false-positive reactions in the CDC *Leishmania* IIF. Because *T. cruzi* is enzootic in domestic dogs and wild canids in some areas of the United States where foxhounds were tested, all samples that yielded *Leishmania* IIF titers >128 were tested in the radioimmunoprecipitation assay (RIPA) for *T. cruzi* at the University of Iowa (*12**,**13*). Sera that gave positive results in both tests were considered positive for *T. cruzi* infection because *Leishmania* antibodies do not give false-positive reactions in the *T. cruzi* RIPA. A group of sera that yielded *Leishmania* spp. IIF titers <128 were randomly selected from foxhounds that were kenneled in southern states (where *T. cruzi* occurs enzootically in wildlife) to further assess the prevalence of *T. cruzi* infection. We defined a confirmed case of *Leishmania* infection as being culture-positive for *L. infantum*, regardless of antibody titer. A probable case was defined as *Leishmania* IIF titer >128 with a negative RIPA for antibodies to *T. cruzi*.

Selected isolates of *Leishmania* spp. cultured from foxhounds were shipped for subtyping and zymodene analysis to the Istituto Superiore di Sanità in Rome, Italy. Montpelier Centre nomenclature for the identification of agents of human leishmaniases was used to classify the organisms.

## Results

From April 2000 to December 2003, >20,000 serum samples were collected from >12,000 canines and submitted to CDC for antibody testing. The dogs ranged in age from 2 months to 13 years. Foxhounds, basset hounds, and beagle hounds represented 91.7%, 2.4%, and 1.3% of the population, respectively. The remainder (4.6%) included >50 other breeds of dogs, foxes, and coyotes.

MFHA-registered fox-hunting clubs are widely dispersed in the eastern half of the United States and Canada, and fewer are located in western states. Of the 210 kennels or hunt clubs that participated in this study, only 29 (14%) were located in states west of the Mississippi River. In contrast, 69 (33%) are located in the 3 states of Pennsylvania, Maryland, and Virginia.

A total of 12,411 dog serum samples from throughout the United States and Canada were submitted to CDC in the first round of sample collection. The distributions of IIF titers in the initial and subsequent rounds of serologic testing are shown in Table 1. Each subsequent round of testing was less comprehensive than the preceding rounds as a result of financial constraints related to collecting blood samples dogs shipping specimens as well as waning interest of owners.

**Table 1:** Distribution of serum *Leishmania* antibody titers in kenneled hunting dogs, United States and Canada, 2000–2003

Serosurvey	IF titer*					
	16	32	64	128	256	>512
First serosurvey (n = 12,411)						
Cumulative no. seroreactive	1,667	736	267	190	133	81
Seroprevalence (%)	13.4	5.9	2.2	1.5	1.1	0.7
Second serosurvey (n = 4,614)						
Cumulative no. seroreactive	1,033	511	195	134	99	62
Seroprevalence (%)	22.4	11.1	4.2	2.9	2.1	1.3
Third serosurvey (n = 1,493)						
Cumulative no. seroreactive	438	211	96	67	52	40
Seroprevalence (%)	29.3	14.1	6.4	4.5	3.5	2.7
Fourth serosurvey (n = 792)						
Cumulative no. seroreactive	262	141	79	58	51	38
Seroprevalence (%)	33.1	17.8	10.0	7.3	6.4	4.8
Fifth serosurvey (n = 571)						
Cumulative no. seroreactive	149	91	58	50	42	33
Seroprevalence (%)	26.1	15.9	10.2	8.8	7.4	5.8
Sixth serosurvey (n = 421)						
Cumulative no. seroreactive	115	76	50	42	37	29
Seroprevalence (%)	27.3	18.1	11.9	10.0	8.8	6.9

*IIF, indirect immunofluorescent assay.

**Figure 1:**
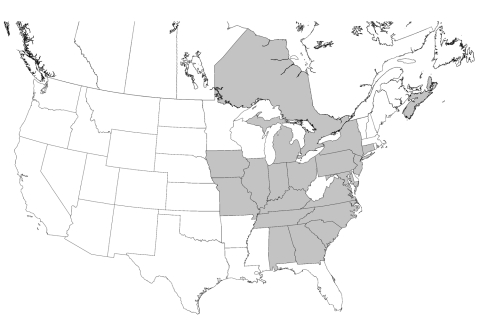
Distribution of hunt clubs with confirmed cases of visceral leishmaniasis, United States and Canada. States in which hunt clubs or kennels had >1 dog infected with *Leishmania infantum* are shaded. *Leishmania*-positive foxhounds were also found in Nova Scotia and Ontario.

**Figure 2:**
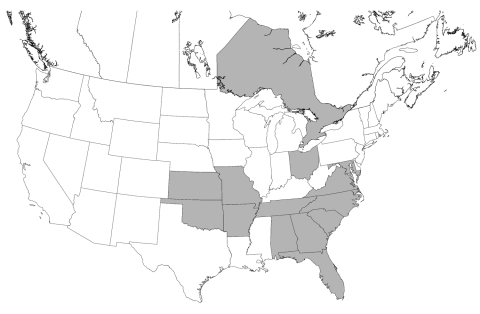
Distribution of hunt clubs with *Trypanosoma cruzi*–positive hounds, United States and Canada. States in which hunt clubs or kennels had >1 dog infected with *T. cruzi* are shaded. A *T. cruzi*–positive hunt club was also found in Ontario.

Infection with *Leishmania* spp. was confirmed in foxhounds from 58 hunt clubs or kennels in 18 states and 2 Canadian provinces (Table 2 and Figure 1). The distribution of *T. cruzi*–infected kennels is shown in Table 3 and Figure 2.

**Table 2:** Distribution of participating foxhound hunt clubs or kennels showing number of hunt clubs with hounds infected with *Leishmania* spp.

State or province	Total hunt clubs tested/positive hunt clubs* (%)
Alabama	4/2 (50)
Arkansas	1/0
Arizona	1/0
British Columbia	1/0
California	4/0
Colorado	3/0
Connecticut	3/2 (66.7)
Florida	6/0
Georgia	6/1 (16.7)
Iowa	2/1 (50)
Illinois	8/4 (50)
Indiana	2/1 (50)
Kansas	2/0
Kentucky	5/2 (40)
Maryland	17/3 (17.6)
Massachusetts	4/0
Michigan	3/2 (66.7)
Minnesota	1/0
Mississippi	2/0
Missouri	3/1 (33.3)
Montreal	1/0
North Carolina	10/3 (30.0)
Nebraska	2/0
Nevada	1/0
New Hampshire	2/0
New Jersey	5/1 (20)
New Mexico	2/0
New York	10/1 (10)
Nova Scotia	1/1
Ohio	7/3
Oklahoma	11/0
Ontario	9/5 (55.6)
Pennsylvania	22/3 (13.6)
South Carolina	7/1 (14.3)
Tennessee	9/1 (11.1)
Texas	9/0
Virginia	32/12 (37.5)
Vermont	1/0
Washington	1/0

*Positive hunt clubs are defined as those that contained >1 positive dog.

**Table 3:** Distribution of participating foxhound hunt clubs or kennels showing number of hunt clubs with hounds infected with *Trypanosoma cruzi*.

State or province	Total hunt clubs tested/positive hunt clubs* (%)
Alabama	4/2 (50)
Arkansas	1/1 (100)
Arizona	1/0
British Columbia	1/0
California	4/0
Colorado	3/0
Connecticut	3/0
Florida	6/3 (50)
Georgia	6/1 (16.7)
Iowa	2/0
Illinois	8/0
Indiana	2/0
Kansas	2/1 (50)
Kentucky	5/0
Maryland	17/3 (17.6)
Massachusetts	4/0
Michigan	3/0
Minnesota	1/0
Mississippi	2/0
Missouri	3/1 (33.3)
Montreal	1/0
North Carolina	10/2 (20)
Nebraska	2/0
Nevada	1/0
New Hampshire	2/0
New Jersey	5/0
New Mexico	2/0
New York	10/0
Nova Scotia	1/0
Ohio	7/1 (14.3)
Oklahoma	2/1 (50)
Ontario	9/1 (11.1)
Pennsylvania	22/0
South Carolina	7/2 (28.6)
Tennessee	9/3 (33.3)
Texas	9/0
Virginia	32/4 (12.5)
Vermont	1/0
Washington	1/0

*Positive hunt clubs are defined as those that contained >1 positive dog.

Collections of multiple serum specimens from individual hounds during the course of the investigation allowed detection of seroconversion over time (Table 4). Seroprevalence in each subsequent round of testing was artificially skewed toward higher values as a result of selective and repeated submission of samples drawn from previously seropositive hounds.

**Table 4:** Positive seroconversion to *Leishmania* spp. or *Trypanosoma cruzi* in kenneled hunting dogs, United States and Canada, 2000–2003*

Characteristic	2000	2001	2002	2003
No. samples tested	12,446	5,487	1,208	1,306
No. new *Leishmania*-positive samples	33	49	9	2
No. new *T. cruzi*–positive samples	6	14	1	0

*Numbers do not include hounds surveyed at the index hunt club in New York.

Demonstration or isolation of *Leishmania* spp. was attempted on blood and tissue specimens submitted by dog owners or their veterinarians from 185 dogs. Tissue specimens for diagnostic culture were collected and submitted to Walter Reed Army Institute of Research or to CDC. Specimens from 62 (33.5%) of 185 hounds were culture-positive. Unexpectedly, 7 (3.8%) positive cultures were from hounds that had *Leishmania* IIF titers <64 (below the positive cutoff titer).

Isolates from 46 foxhounds were sent to the Reference Centre in Rome, Italy, for zymodeme analysis. The isoenzyme characterization showed the agent isolated from 46 foxhounds to be *Leishmania infantum* zymodeme MON1.

Sera were tested from numerous other breeds of dogs from many states, including shelter dogs from Dutchess County, New York, and Orange County, Virginia, where infection had been confirmed in foxhounds and wild canids (n = 286) collected in the southeastern United States. All of these samples were negative in the *Leishmania* spp. IIF (Table 5). None of the samples from dogs were positive in the *T. cruzi* RIPA; however, *T. cruzi* infection was detected serologically in 2 wild canids.

**Table 5:** Distribution of *Leishmania* antibody titers in pet dogs, shelter dogs, and wild canids, 2001–2002

Animal	Titer						
	<16	16	32	64	128	256	>512
Pet dogs* (n = 709)	706	3	0	0	0	0	0
Shelter dogs,† Dutchess County (n = 74)	71	2	1	0	0	0	0
Shelter dogs,† Orange County (n = 55)	53	2	0	0	0	0	0
Wild canids‡ (n = 291)	286	2	2§	0	1§	0	0

*Samples from pet and sporting dogs submitted to the Centers for Disease Control and Prevention from numerous states for leishmaniasis testing to fulfill entry requirements for countries that require it. †Samples from dogs retained in county animal shelters. ‡Samples from wild canids collected in the southeastern United States included. Species included red fox (*Vulpes vulpes*, n = 158), gray fox (*Urocyon cinereoargentus*, n = 51), and coyote (*Canis latrans*, n = 82). §1 of the 2 canids with titer of 32 and the canid with titer of 128 were *Trypanosoma cruzi* RIPA-positive.

Serum samples obtained from 158 persons associated with foxhounds were tested by the *Leishmania* spp. IIF. None of the persons who provided blood samples reported signs or symptoms suggestive of leishmaniasis, and all of the samples gave titers below the positive cutoff.

## Discussion

Our survey of foxhounds, other breeds of dogs, and wild canids showed that canine visceral leishmaniasis is enzootic in 18 US states and 2 Canadian provinces. Newly seroconverted cases were detected each year during the investigation (2000–2003), which indicates that active transmission of the parasite continues.

Data from this investigation indicate that autochthonous infection in canines is predominantly limited to foxhounds. Increased susceptibility of this breed is possible, although comparative infection studies have not been carried out. Several factors concerning management of foxhounds may favor transmission: the gregarious nature of the breed, concentration of large numbers of dogs within hunt clubs, and management practices, such as intrastate and interstate movement and interbreeding and exchange of dogs between hunt clubs. More than 200 MFHA-registered kennels in the United States and Canada house >12,000 hounds. Most of these dogs are foxhounds that travel frequently and extensively to participate in field trials, for trading and interbreeding between kennels and hunt clubs, and to be shown at dog shows. These management practices appear to have enabled leishmanial infection to spread widely in this breed throughout the eastern United States and Canada.

Testing for *Leishmania* antibodies in pet dogs from numerous states and in shelter dogs in New York and Virginia failed to identify any *Leishmania*-positive animals. Similarly, no antibody evidence of *Leishmania* infection was detected among 291 wild canids taken from various states where infections in kenneled foxhounds were diagnosed. By contrast, 2 wild canids were infected with *T. cruzi*. Taken together, these findings suggest that *Leishmania* infection in foxhounds is transmitted from dog to dog.

Antibody testing of 158 humans associated with infected foxhounds did not identify any seropositive persons, nor have autochthonous cases of visceral leishmaniasis in humans been diagnosed in North America. Transmission among foxhounds and other breeds appears to be limited to direct dog-to-dog mechanisms; this assumption is supported by lack of apparent transmission to humans. The fact remains, however, that a zoonotic disease has been introduced into the canine population in the United States and Canada. Visceral leishmaniasis in humans has variable onset and manifestations, and delay or misdiagnosis in areas where the disease is not endemic is common (*14*).

In regions of transmission outside North America, canine visceral leishmaniasis, caused by *L. infantum* (*L. chagasi* in the New World), is transmitted by sandfly vectors (*1**–**3*). Although sandflies indigenous to North America have not been implicated in transmission of visceral leishmaniasis, 4 species of North American sandflies of the genus *Lutzomyia* are mammalian feeders. *Lutzomyia anthorphora* and *Lu. diabolica* are found in Texas, and *Lu. cruciata* is found in Florida and Georgia (*15*). *Lu. shannoni* has been identified in Alabama, Arkansas, Delaware, Florida, Georgia, Louisiana, Mississippi, North Carolina, South Carolina, and New Jersey (*15*). The range of *Lu. shannoni* overlaps the locations of many of the hunt clubs in which we found *Leishmania*-infected dogs.

Experimental studies showed that *Lu. shannoni* became infected with *L. infantum* after feeding on *L. infantum*–infected dogs (*16*). Investigators hypothesized that these insects were competent vectors and could initiate new enzootic cycles of *Leishmania* transmission in areas where infected animals were introduced (e.g., North America) (*16*). As the reservoir of infection in canine hosts becomes larger and more dispersed, the possibility increases that conditions will lead to exposure of competent vectors and subsequent vectorborne transmission.

The data collected in this investigation and the apparent absence of active vector transmission suggest that spread of infection in foxhounds and other dogs occurred by direct dog-to-dog transmission. Infected dogs, including those in preclinical or subclinical stages, can be reservoirs of infection for uninfected animals. Possible modes of direct dog-to-dog transmission include biting, reusing needles for injections, blood transfusions, and breeding. Dog bites and other abrasions and lacerations occur commonly among working and kenneled foxhounds, which may potentiate exchange of body fluids between hounds. Blood transfusion transmission from infected dogs was documented in a clinical study at the University of Pennsylvania (*17*). Congenital transmission from infected, pregnant female foxhounds to their pups was observed by owners and reported during the course of our investigation. Transplacental transmission in an experimentally infected beagle was recently described by Rosypal et al. (*18*). Breeding that results in transplacental infection of litters may be the most important mechanism of transmission among foxhounds, which explains why this infection is limited to foxhounds even in situations in which foxhounds are housed with beagles and basset hounds (data not shown).

Cross-reacting anti–*T. cruzi* antibodies give false-positive results in the CDC *Leishmania* IIF, but anti-*Leishmania* antibodies do not give false-positive results in the *T. cruzi* RIPA (*12**,**13*). We took advantage of this difference by doing RIPA testing on samples from 413 hounds that had *Leishmania* IIF titers >32. Eighty-six (21%) of these specimens gave positive results, which indicates that the hounds were infected with *T. cruzi*. The remaining 326 (78.9%) that were negative by RIPA were considered to be infected with *L. infantum*. Dogs with dual reactivity to the *Leishmania* and *T. cruzi* antibody assays may have been infected with both protozoal agents; however, we could not confirm this possibility, and we believe that the probability is low and inconsequential to this study.

Previous reports across several decades have indicated that *T. cruzi* is enzootic in domestic dogs and wild canids in the southern United States (*12**,**13*), a consequence of the sylvatic cycle of *T. cruzi* that involves triatomine insects and various mammalian hosts. Dogs are believed to become infected by exposure to infected vectors or by eating infected wild mammals, such as armadillos, raccoons, opossums, and wood rats. In contrast to the situation with *Leishmania* spp., direct dog-to-dog transmission of *T. cruzi* is likely less frequent, although congenital transmission and transmission through blood transfusion may help maintain the parasite in dog populations.

The widespread geographic distribution and prevalence of *T. cruzi* infection in hounds reported here expand our understanding of this highly pathogenic parasite in dogs in the United States. *T. cruzi* causes severe clinical manifestations in dogs (*13*), as it does in humans (*19*). In view of these findings, veterinarians in enzootic areas should include *T. cruzi* infection in the differential diagnosis of dogs with unexplained cardiac disease. Unfortunately, transmission of *T. cruzi* to dogs cannot be prevented other than by effective vector control and not allowing dogs to run unsupervised, and no curative treatment for *T. cruzi* infection is available.

Participation in this investigation by foxhound owners was voluntary, and the loss to follow-up of many animals after the initial serosurvey detracted from our ability to comprehensively assess the incidence and geographic extent of this infection. Although new cases of leishmaniasis and trypanosomiasis were discovered in the sequential serosurveys, we could not calculate the incidence of these parasitoses among exposed foxhounds. As a result of decreasing compliance, the second and subsequent serosurveys were less comprehensive than the initial serosurvey, despite our request that all hounds be retested. Although serologic testing at CDC was offered at no charge, decreasing participation may have been caused by the cost of specimen collection and shipment or by declining interest.

The distribution of canine visceral leishmaniasis in the United States and Canada was determined by using defined case definitions based on confirmatory laboratory data. Data were analyzed with strict adherence to case definitions. Twelve suspected cases were excluded from analysis because they did not meet the case definitions.

Preliminary recommendations to limit spread of *Leishmania* infection were communicated to foxhound owners in 2000. CDC recommended a moratorium on exchange of hounds between hunt clubs and commingling of hounds from different hunt clubs for at least 1 year. Intra-hunt club recommendations to limit transmission were also suggested.

Recommendations to segregate infected animals, suspend dog shows and hunting for clubs or kennels with dogs with leishmaniasis, and avoid commingling and interbreeding of animals between hunt clubs were implemented initially by most of the hunt clubs or kennels. Nevertheless, cooperation appears to have been short-lived. In 2001, after 1 year of general adherence to the recommendations, hunting and commingling resumed and continues in most hunt clubs and kennels. Factors that led to this decision in the hunting community included the belief that the disease was not a threat to the well-being of the animals or the persons involved. The perceived low illness and death rates associated with leishmaniasis in this canine population and the inconvenience and cost of recommended control measures may have also led some hunt club owners to ignore them.

The presence of *L. infantum*–infected dogs in areas in the United States and Canada where sandflies have not been identified is now well established. Sandflies may exist in these areas but have not yet been identified, or another arthropod species may be responsible for *Leishmania* transmission. The mechanisms by which canine visceral leishmaniasis can be transmitted among dogs in the absence of vectors warrant further investigation. Because most leishmanial infections in dogs appear limited to foxhounds, breed susceptibility and breeding-associated transmission mechanisms should be further examined.

The widespread presence of *L. infantum* infection in foxhounds in North America represents potential public and canine health threats that should be addressed by further investigation and control measures. Based on findings from this investigation and what is known about the biologic behavior of *Leishmania* spp., the following recommendations should be considered to manage and control leishmaniasis in foxhound kennels. All dogs in a hunt club or kennel should be tested serologically to identify those infected with the parasite. All dogs considered for breeding should be >2 years of age and should be evaluated serologically for *Leishmania* infection. Dogs with confirmed or suspected leishmaniasis should be excluded from breeding programs. Dogs with positive serum *Leishmania* titers should have cultures performed to confirm infection status. All dogs that are confirmed to have *Leishmania* infection should be euthanized. Dogs with borderline or suspicious titers should be considered as possibly infected and retested in 3–6 months for further assessment. All dogs that have positive results when tested for *Leishmania* antibodies and have either not had specimens cultured or had culture-negative results should be tested for specific antibodies to *T. cruzi*. The recent report of an effective vaccine for canine leishmaniasis (*20*) suggests an additional tool to prevent and eliminate this infection in North America, although further research is necessary to define its role in a prevention strategy. The effectiveness of control measures must be monitored by surveillance of foxhounds and associated dog breeds by using sensitive diagnostic screening methods.
